# Biologic and Small Molecule Therapy in Atopic Dermatitis

**DOI:** 10.3390/biomedicines12081841

**Published:** 2024-08-13

**Authors:** Mahek Shergill, Barinder Bajwa, Orhan Yilmaz, Karishma Tailor, Naila Bouadi, Ilya Mukovozov

**Affiliations:** 1 Michael G. DeGroote School of Medicine, McMaster University, Hamilton, ON L8P 1H6, Canada; mahek.shergill@medportal.ca; 2 Faculty of Medicine, University of British Columbia, Vancouver, BC V6T 1Z3, Canada; bbajwa@student.ubc.ca; 3 College of Medicine, University of Saskatchewan, Saskatoon, SK S7N 5E5, Canada; orhan.yilmaz@usask.ca; 4 Faculty of Medicine, University of Ottawa, Ottawa, ON K1H 8M5, Canada; ktail064@uottawa.ca; 5 Faculty of Medicine, Laval University, Quebec, QC G1V 0A6, Canada; naila.bouadi.1@ulaval.ca; 6 Toronto Dermatology Centre, Toronto, ON M3H 5Y8, Canada

**Keywords:** AD, Janus kinase inhibitors, JAK1, JAK2, JAK3, TYK2, dupilumab

## Abstract

Atopic dermatitis is a chronic inflammatory dermatosis characterized by pruritic, scaly, erythematous lesions. Its incidence varies but is estimated to be approximately 20% in children and between 7 and 14% in adults, with variation amongst countries. It is a multifactorial condition, with a complex interplay between genetic, immunological, and environmental factors. Research into the inflammatory response has identified new therapeutic targets that work to reduce inflammation and subsequently reduce flares. This study explores existing therapeutic agents for atopic dermatitis as well as newer therapies such as biologics and small molecules, drawing upon each agent’s mechanism of action, relevant landmark clinical trials, efficacy, and safety profile. Current therapies include emollients, corticosteroids, cyclosporine A, calcineurin inhibitors, phototherapy, and methotrexate. Biologics described include dupilumab, tralokinumab, lebrikizumab, nemolizumab, and rocatinlimab. Small molecules inhibitors include Janus kinase inhibitors, phosphodiesterase 4 inhibitors, transient receptor potential vanilloid subfamily V member 1 antagonist, and aryl hydrocarbon receptor antagonist.

## 1. Introduction

Atopic dermatitis (AD), commonly referred to as eczema, is an inflammatory skin condition that presents in childhood but can persist into adulthood [1]. The global prevalence of AD is estimated to be between 15 to 20% among children and 10% among adults, with studies documenting an increasing prevalence over the course of the past several decades [1]. It is characterized by the presence of pruritic, erythematous, scaly lesions that are commonly found over flexural areas; however, clinical presentation varies among different races and ethnic groups [2]. It is associated with asthma, and allergic rhinitis as part of the atopic triad [2]. In clinical trials, AD severity is typically scored using the Eczema Area and Severity Index (EASI), which is graded on a seven-point scale in four areas of the body based on four signs of clinical severity, namely, erythema, edema, excoriation and lichenification, allowing for a maximum score of 72 [3]. Another commonly used scale is the scoring AD scale (SCORAD), which assesses severity based on six clinical signs, namely, erythema, edema, oozing/crust, excoriation, lichenification, and dryness, on a four-point scale, in addition to patient reported sleep loss and pruritus, allowing for a maximum score of 103 [3].

## 2. Current Therapies

There are several therapies available for AD, including emollients, corticosteroids, cyclosporine A, calcineurin inhibitors, phototherapy, and methotrexate.

Emollients are a fundamental component in the multifaceted approach to managing AD. Emollients may be found in vehicles as creams, ointments, and lotions. Each variation offers unique formulations to cater to varying degrees of severity and patient preferences. With regular application, emollients may be used prophylactically, and as a supportive measure to maintain the skin barrier [4].

AD is denoted by an impaired skin barrier function, subsequently causing increasing transepidermal water loss and susceptibility to irritants [5]. The mechanism of action revolves around their ability to moisturize and restore the skin barrier, reducing itching and irritation, preventing allergen penetration, and enhancing topical treatments [5]. This works to maintain optimal skin hydration, where dehydrated skin exacerbates symptoms. Research studies examining the use of emollients in cases of mild AD demonstrate benefits such as reduction in SCORAD, improvement in sleep quality and pruritus, and improvement in flares [6]. For example, one study found that an emollient with colloidal oatmeal demonstrated that 80% of flares had improved or cleared at week 4, a lower incidence of flares, and shorter median time to flares [6]. The relative ease of integrating emollients into patients’ lifestyle makes it a viable therapeutic option for milder forms of AD.

Corticosteroids are a cornerstone in the pharmacological treatments for AD. Corticosteroids are anti-inflammatory agents that exert their effect by modulating immune response and mitigating symptoms like itching, redness, and inflammation [7].

Corticosteroids bind to cytoplasmic receptors in target cells, forming complexes that enter the nucleus and subsequently block inflammatory gene transcription [8]. They block inflammatory protein mediators, such as cytokines and chemokines, contributing to the downregulation of inflammation [9]. Furthermore, corticosteroids can induce vasoconstriction and decrease vascular permeability. Reducing blood flow to the affected area and reducing leakage of fluid from blood vessels to the surrounding tissues decreases redness and edema-associated inflammation [10,11].

Cyclosporine A (CsA), a systemic immunosuppressant, may be used in severe AD [12]. CsA, a calcineurin inhibitor, effectively modulated T cell activation, reducing the inflammatory cascade of AD [12]. Notably, this approach is used for cases where other interventions are insufficient or impractical due to the limitations posed by the potential side effects, such as nephrotoxicity, hypertension, and increased susceptibility to infections [12].

More specifically, CsA primarily targets T lymphocytes by binding to cyclophilin, a cellular protein forming a complex that inhibits the activity of calcineurin, an essential component in the activation of T cells [13]. By preventing the activation and proliferation of T cells, CsA inhibits the release of proinflammatory cytokines including interleukins (Ils) and tumour necrosis factor-alpha [13]. Ultimately, CsA modulates the immune response and mitigate inflammation in the skin.

Calcineurin is a phosphatase enzyme critical for the activation of T cells [14]. With T cell receptor stimulation, calcineurin dephosphorylates the nuclear factor of activated T cells (NFATs), allowing its translocation to the nucleus [14]. NFAT activation leads to the transcription of various cytokines which contribute to the inflammatory response [14]. Thus, calcineurin inhibitors block the activity of calcineurin, disrupt the signaling pathway and inhibit the production of proinflammatory cytokines, such as interferon-gamma and tumor necrosis factor-alpha [14]. Similar to CsA, these inhibitors stabilize mast cells, preventing the release of mediators and reducing pruritus [14]. Topical calcineurin inhibitors, including tacrolimus and pimecrolimus, are typically a long-term alternative to corticosteroids [15,16]. Calcineurin inhibitors are valuable for AD found in the face and intertriginous regions, especially as the risk of corticosteroid-induced skin atrophy is heightened [15].

Phototherapy, also known as light therapy, is a non-pharmacological approach to managing AD, which is achieved by exposing the skin to controlled doses of ultraviolet light [17]. Ultraviolet light modifies the activity of immune cells, modulates cytokine production, and promotes apoptosis of inflammatory cells [17]. Both ultraviolet U (UVA) and ultraviolet B (UVB) phototherapy have demonstrated efficacy in suppressing immune responses, particularly T lymphocytes [17].

UVA and UVB phototherapy may be administered in various forms, including narrowband UVB (nbUVB) and broadband UVB (bbUVB) [18]. nbUVB utilizes a specific wavelength (311–313 nm) of light to target the skin and minimize side effects [17,18,19]. This method is considered effective in treating AD with fewer side effects compared to bbUVB [17]. While bbUVB may be effective, it has a higher risk of erythema compared to nbUVB [17].

Phototherapy is a modality often considered for those unresponsive to conventional treatments or those with widespread involvement of AD. The use of phototherapy necessitates careful consideration of potential side effects, including premature skin aging [17,19]. Limitations of phototherapy include cost, accessibility and patient compliance given that administration is spaced over time [17].

Methotrexate is a systemic immunomodulator commonly used in cases of moderate to severe AD [20]. For example, a study investigating the use of methotrexate in children found that therapeutic efficacy was achieved, and that the mean Investigator Global Assessment (IGA) improvement was from 4.3 to 2.8 after 3 to 5 months of administration, and to 1.9 after 10 months [20]. The study also described that the Children’s Dermatology Life Quality Index initially improved from 14.4 at baseline to 7.5 at 3 to 5 months of treatment and decreased to 6.6 at the 10-month mark [20]. Importantly, some of the risks of long-term methotrexate administration include the development of fatty liver and hepatic fibrosis, making routine patient monitoring critical [21].

**Figure 1:**
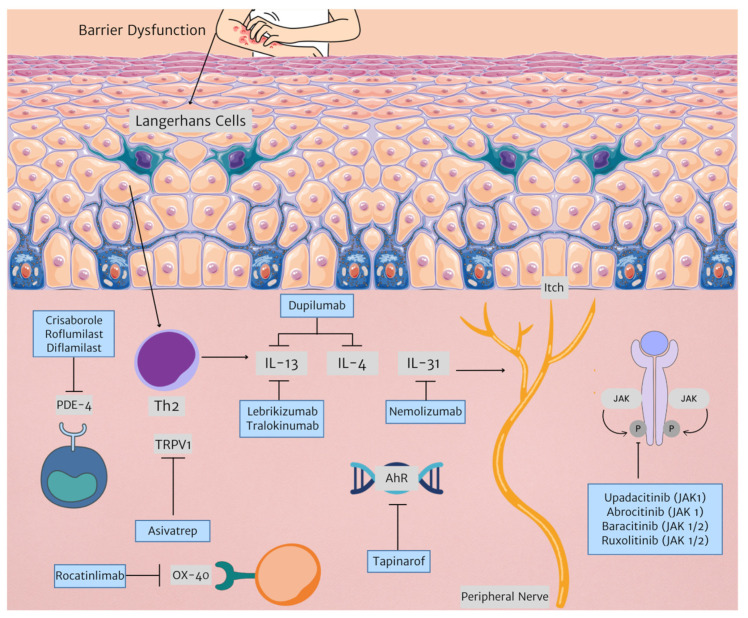
New therapies in AD treatment and their respective targets. (Triangle arrowhead representing next step in mechanism. Arrow with horizontal line represents inhibition. Abbreviations: AhR: aryl hydrocarbon receptor; IL: interleukin; JAK: janus kinase; PDE-4: phosphodiesterase 4, Th2: T helper 2, TRPV1: transient receptor potential vanilloid subfamily V member 1).

Current therapies are an effective treatment option for many patients with AD. However, new therapeutic targets allow for more tailored management. Figure 1 illustrates newer therapies developed for AD treatment as outlined in this paper.

## 3. Phosphodiesterase 4 Inhibitors

### 3.1. Crisaborole

Crisaborole, a boron-based compound, is a selective nonsteroidal inhibitor of phosphodiesterase 4 (PDE4), effectively reducing inflammation in AD. It works by inhibiting the breakdown of cyclic adenosine monophosphate, which suppresses various inflammatory cytokines like tumor necrosis factor-alpha, ILs nuclear factor-kappa B, and prostaglandin E2 [22,23,24]. This mechanism helps mitigate inflammatory processes in AD, including type 1 helper T cell, Th2, and Th17/Th22 axes [25]. Additionally, crisaborole is rapidly metabolized into inactive metabolites resulting in limited systemic exposure, thus avoiding the potential side effects often associated with oral PDE4 inhibitors [26].

In clinical trials, crisaborole has shown significant efficacy in treating AD. It is approved to be used in both adults and children 3 months and older. In a phase IV study, about 30% and 47% of patients achieved IGA clear or almost clear status by day 29, respectively [27]. Common side effects included application site pain, discomfort, and erythema. Two phase III trials (AD-301 and AD-302) further demonstrated its effectiveness, with success rates of 32.8% vs. 25.4% in AD-301 and 31.4% vs. 18.0% in AD-302 for crisaborole vs. vehicle groups [28].

A phase IIa study showed that 68% of patients experienced a significant decrease in the AD Severity Index score after 28 days of treatment, with improvements in symptoms like pruritus, erythema, and lichenification [23]. A retrospective study on crisaborole 2% highlighted application site pain as a key adverse event, with a higher incidence of pain (31.7%) compared to phase III clinical trials (4.4%) [29]. Pain typically occurred within minutes after application, being more prevalent with facial application [29]. Clinical trials have underscored crisaborole’s efficacy in relieving AD symptoms, with improvements in skin clarity and quality of life measures [30,31,32,33]. Its favorable safety profile is attributed to its rapid metabolism and minimal systemic exposure, especially in children [25].

### 3.2. Roflumilast

Roflumilast, a second-generation PDE4 inhibitor, has a reduced risk of causing emesis compared to its predecessors [30]. It is approved in adults and children 6 years and older. A phase II trial with 40 moderate AD patients compared roflumilast 0.5% cream to a vehicle cream, and no notable differences were observed in SCORAD, transepidermal water loss, or pruritus scores [22]. Mild to moderate side effects like pain at the application site, increased liver enzymes, and nasopharyngitis were reported [22].

Oral roflumilast can cause severe side effects like nausea, vomiting, and psychiatric issues. Therefore, topical application is recommended to reduce these risks [34].

### 3.3. Difamilast

Difamilast, a PDE4 inhibitor, has been shown to reduce cytokine production associated with AD [35]. It has yet to be approved in the United States. In a key phase III study, a 1% difamilast ointment was significantly better compared to a placebo in improving the IGA score by week 4 [35]. Improvements of 50%, 75%, and 90% were observed in the EASI score [35].

A separate phase III study highlighted the effectiveness of difamilast, showing higher success rates in the EASI score for both 0.3% and 1% difamilast groups compared to the placebo [36]. This difference was consistent from the first to the fourth week [36]. Nasopharyngitis, impetigo, and worsening AD were the most common side effects, indicating a positive safety profile for difamilast in treating pediatric AD patients [36].

## 4. Overview of Biologics

Biologics represent a new frontier in the treatment of AD. They are antibodies that work by targeting specific components of the immune system (e.g., cytokines or receptors) involved in the inflammatory response. By blocking these cytokine/receptor pathways, biologics help modulate the immune system’s hyperactive response seen in AD, ultimately reducing skin inflammation, redness, and itching.

### 4.1. Dupilumab

Dupilumab, a human monoclonal IgG4 antibody, works by inhibiting IL-4 and IL-13 signaling through competitive binding to the shared subunit of the IL-4 receptor subunit [37]. Dupilumab is approved for the treatment of AD in adults and children 6 months and older.

The efficacy and safety of dupilumab has been studied in numerous randomized control trials [38]. SOLO 1 and SOLO 2 enrolled adults with an IGA score of 3 or 4 [39]. In SOLO 1, 38% reached an IGA score of 0 or 1 using dupilumab every other week, while in SOLO 2, 36% achieved this reduction [39]. A higher proportion of patients receiving dupilumab had a 75% improvement on EASI by week 16 compared to placebo [39].

In the LIBERTY AD CHRONOS trial, adult patients with moderate to severe AD and an inadequate response to topical corticosteroids (TCSs) received dupilumab either weekly or biweekly alongside TCSs [40]. By week 16, 39% of patients on weekly dupilumab and 39% on biweekly dupilumab in combination with TCSs achieved an IGA improvement of 0 or 1 [40]. This contrasted with 12% of patients who received a placebo with TCSs [40]. The combination of dupilumab with TCSs notably improved patient-reported symptoms of AD, including a positive impact on sleep among affected individuals [40].

Similarly, LIBERTY AD ADOL is a randomized control trial that focuses on the use of dupilumab monotherapy in adolescents from 12 to 18 years old with AD [41]. This trial also demonstrated substantial improvement in EASI-75 and peak pruritus numerical rating scale [41]. Side effects included paradoxical head and neck erythema, ocular complications, new-onset psoriasis, arthritis, and alopecia [42,43].

### 4.2. Tralokinumab

Tralokinumab, a fully human IgG4 monoclonal antibody, works by targeting and binding strongly to interleukin-13 (IL-13), a cytokine involved in the inflammatory processes associated with AD [44,45]. By blocking the interaction of IL-13 with its receptors, tralokinumab helps to inhibit the signaling pathways responsible for the inflammation underlying AD [45].

Tralokinumab is approved in adults and children 12 years and older. ECZTRA 1 and ECZTRA 2, both phase III trials, randomized adults with moderate to severe AD to receive tralokinumab 300 mg every two weeks or a placebo [46]. Tralokinumab showed higher IGA 0 or 1 rates and EASI 75 at week 16 compared to placebo [46].

Additionally, ECZTRA 6, a randomized control trial focused on patients aged between 12 to 17 years old, described statistically significant differences in IGA scores 0 or 1 at week 16 in the tralokinumab arm versus placebo [47].

While tralokinumab has shown to improve AD in patients, this biologic is associated with adverse events such as upper respiratory tract infection, conjunctivitis, skin infection, pruritus, and headache [47,48].

### 4.3. Lebrikizumab

Lebrikizumab, a monoclonal antibody, works by directly targeting IL-13, a crucial element in the inflammatory process associated with AD [49]. By specifically binding to IL-13, lebrikizumab blocks its interaction with immune cell receptors, interrupting the signaling pathway responsible for inflammation, and offering potential relief by minimizing inflammation [49]. Lebrikizumab is not approved in the United States.

Two randomized 52-week trials, ADvocate1 and ADvocate2, were also executed to assess the safety and effectiveness of lebrikizumab as a monotherapy for moderate to severe AD in both adults and adolescents [50]. In both trials, a notably greater proportion of patients receiving lebrikizumab achieved an IGA score of 0 or 1, and a reduction of ≥2 points from baseline by week 16 compared to those in the placebo group [50]. In trial 1, 43.1% of patients in the lebrikizumab group achieved this response, vs. 12.7% in the placebo group [50]. Trial 2 similarly demonstrated a substantial difference, with 33.2% of lebrikizumab-treated patients achieving the primary outcome response compared to 10.8% in the placebo group [50].

Adverse events noted on patients treated with lebrikizumab were conjunctivitis, dermatitis exacerbation, and skin infection [50].

### 4.4. Nemolizumab

Nemolizumab is a fully human monoclonal antibody targeting the interleukin-31 receptor A (IL-31RA). IL-31RA is a key component in the itch–scratch cycle associated with AD [51]. IL-31 is a cytokine involved in the pathogenesis of AD, which binds to IL-31RA on sensory neurons, triggering pruritus and perpetuating the itch –scratch cycle [51,52]. Through binding to IL-31RA, nemolizumab disrupts the pathway, attenuating the itching and decreasing overall disease severity. Nemolizumab has yet to be approved in the United States.

There have been several clinical trials conducted on nemolizumab within the context of AD. In particular, XCIMA, a phase II trial, described significant changes on the pruritus visual analogue scale, at −43.7% in the 0.1 mg/kg group, −59.8% in the 0.5 mg/kg group, and −63.1% in the 2.0 mg/kg group, versus −20.9% in the placebo group at week 12 [53]. Additionally, EASI changes were −23.0%, −42.3%, and −40.9%, respectively, in the nemolizumab groups, versus −26.6% in the placebo group [53].

Although nemolizumab demonstrated a favorable safety profile, common adverse events included mild to moderate injection site reactions and signs of upper respiratory tract infections [52].

### 4.5. Rocatinlimab

OX40 is a costimulatory molecule vastly expressed on activated effector T cells, with a critical role in the differentiation and memory induction of T cells and is prominently expressed in those with AD [54]. Rocatinlimab, is an anti-OX40 antibody, inhibiting and reducing OX40 pathogenic T cells responsible for AD inflammatory responses [54]. By blocking OX40, signals critical for expansion and survival of pathogenic T cells and subsequent generation of memory T cells are prevented, inhibiting inflammation [54]. Rocatinlimab has not been currently approved for AD in the United States.

A multicenter, double-blind, placebo-controlled phase IIb study was performed to evaluate the safety and efficacy. Compared with placebo, significant reductions in EASI score at week 16 were observed in all rocatinlimab groups [54]. Efficacy measures continued to improve after week 16 for all rocatinlimab groups, with the highest responses observed in the 300 mg every 2 weeks group [55].

The most common side effects found during the study included pyrexia, nasopharyngitis, chills, headache, aphthous ulcer, and nausea [54].

## 5. Overview of Small Molecules

### 5.1. Janus Kinase Inhibitors

The mechanism by which cells communicate signals from their exterior to the nucleus involves the Janus kinase and signal transducer and activator of transcription (JAK-STAT) pathway [56]. Within the JAK family of kinases, namely, JAK1, JAK2, JAK3, and TYK2, the intricate orchestration of cytokine-mediated communication is executed [57]. This pathway serves as a critical conduit for numerous inflammatory cytokines and signaling molecules, contributing significantly to autoimmune and autoinflammatory conditions where disease-causing cytokines depend on JAK-STAT signaling to manifest their pathogenic effects [58,59].

The pathogenesis of AD is linked to enhanced Th2 immunity, driven by JAK-STAT signaling downstream of key cytokines such as IL-4, IL-5, and IL-13 [60]. Building on these insights into the underlying mechanisms, JAK inhibitors have emerged as potential therapeutic agents for AD. Their exploration as a treatment modality stems from the recognition of the central role played by JAK-STAT signaling in the pathophysiology of AD [60].

#### 5.1.1. Baricitinib

In 2023, the BREEZE-AD PEDS clinical trials highlighted the potential of baricitinib, a JAK1/JAK2 inhibitor, in pediatric patients with moderate to severe AD, who showed inadequate responses to TCSs [61]. Baricitinib, when administered at a 4 mg dose, showed significant improvement by week 16, where patients had a ≥75% improvement in EASI-75 and a ≥90% improvement in EASI-90 [61]. The most common side effects included abdominal pain, acne, headache, diarrhea, nasopharyngitis, and upper respiratory tract infection [61]. This trial underscored baricitinib effectiveness in improving key disease measures, such as skin inflammation and itch in pediatric patients [61]. Baricitinib has demonstrated safety in the pediatric population, but has yet to be approved for AD.

The BREEZE-AD4 trial in 2022 evaluated baricitinib with background TCS in a similar patient group [62]. The study found that this combination was more effective than placebo with TCSs in achieving EASI 75, at 32% and 17%, respectively, at week 16 [62]. Additionally, it demonstrated superior results compared to placebo, especially in reducing itch, skin pain, and night-time awakenings [62].

#### 5.1.2. Upadacitinib

The AD Up Trial in 2021 investigated oral upadacitinib, a selective JAK1 inhibitor, in combination with TCS [63]. It showed that both 15 mg and 30 mg doses of upadacitinib significantly enhanced treatment outcomes compared to placebo [63]. For instance, by week 16, a significantly higher proportion of patients in both upadacitinib groups achieved EASI-75 compared to the placebo group, specifically 65% for a 15 mg dose and 77% for a 30 mg dose compared to 26% in placebo [63]. Upadacitinib has yet to be approved.

#### 5.1.3. Ruxolitinib

The TRuE-AD trial explored the use of ruxolitinib, a JAK1/JAK2 inhibitor, revealing substantial improvements in disease measures [64]. The trials explored the use of 0.75% and 1.5% ruxolitinib, and the results described treatment success in both compared to vehicle cream by week 8 [64]. Additionally, results showed rapid itch reduction starting within 12 h of the first application of 1.5% ruxolitinib cream [64]. Ruxolitinib has yet to be approved.

#### 5.1.4. Abrocitinib

The JADE MONO 1 and JADE MONO 2 studies in 2020 focused on abrocitinib, a selective JAK1 inhibitor [65,66]. At 12 weeks, patients receiving abrocitinib at dosages of 100 mg and 200 mg demonstrated IGA success, at 28.4% and 38.1%, respectively, versus 9.1% in placebo [65]. Regarding EASI-75, 61.0% in the 200 mg group and 44.5% in the 100 mg group reached this benchmark, compared to just 10.4% in the placebo group [65].

Regarding safety, common side effects included upper respiratory tract infections, nausea, and increased liver enzyme levels [65]. Abrocitinib has been approved for adults and children over the age of 12 for treatment of AD.

## 6. TRPV1 Antagonist

Transient receptor potential vanilloid subfamily V member 1 [TRPV1] is a non-selective cation channel, and is present in various skin cells including keratinocytes, mast cells, and sensory nerves [67]. TRPV1 is activated in the skin lesions associated with AD, worsening pruritus and inflammation [67]. Asivatrep, a TRPV1 antagonist works by enhancing the production of key markers involved in skin differentiation, such as loricrin, filaggrin, involucrin, and certain keratins, leading to a reduction in dermatitis and pruritis [67]. Asivatrep has yet to be approved for AD in the United States.

In a phase III study known as CAPTAIN-AD, the efficacy of asivatrep in treating AD was assessed [67]. The study included patients 12 years and older with mild to moderate AD who were treated with asivatrep cream twice daily or placebo cream for 8 weeks [67]. Of the total, 36% of patients in the asivatrep group achieved an IGA score of 0 or 1 by week 8, compared to 12.8% in the placebo group [67]. Associated side effects were mild and included nasopharyngitis, urticaria, burning sensation, and rhinorrhea.

## 7. Aryl Hydrocarbon Receptor Antagonist

The innovative treatment of AD has recently been advanced through the study of tapinarof, an aryl hydrocarbon receptor antagonist [68]. The efficacy of tapinarof in AD is primarily due to its ability to reduce proinflammatory type 2 cytokine expression and oxidative stress, enhance the expression of skin barrier proteins, and restore skin homeostasis [68,69,70]. Tapinarof is not FDA approved yet.

In a phase II randomized clinical trial, adolescents and adults with AD were assigned to receive tapinarof cream at concentrations of 0.5% or 1%, or a placebo, either once or twice daily for 12 weeks, followed by a 4-week observation period [71]. Notably, the tapinarof cream groups demonstrated numerically higher IGA response rates compared to the vehicle groups from week 2 onwards, with significant improvements in EASI75 scores, except in the 0.5% once-daily group, by week 12 [71].

Tapinarof was predominantly well tolerated; however, side effects included nasopharyngitis, folliculitis, AD exacerbation, upper respiratory tract infections, headaches, acne, and impetigo [72].

## 8. Conclusions

AD is a chronic inflammatory condition with significant prevalence in both children and adults. The psychosocial impacts of AD remain multifaceted, impacting patient’s quality of life substantially. The variety of treatments allows for its chronic course to be managed over time, providing options for patients with mild to severe disease.

The future of atopic dermatitis therapy continues to evolve, with research exploring microbiome changes that substantially influence the course of AD [73]. For example, the development of antimicrobial peptide topicals is being explored to counteract the greater *S. aureus* burden seen in AD and increase microbiome diversity [73]. Similarly, live biotherapeutic products are being investigated in the treatment of AD, such as *Roseomonas mucosa*, a Gram-negative skin commensal positioned to decrease *S. aureus* colonization [74].

Therapeutic agents such as biologics and small molecule inhibitors remain a promising avenue with fewer side effects, but further research is necessary to determine their application in pediatric populations and those with comorbidities. Table 1 consolidates landmark trials for each of the new agents discussed in this paper, in addition to detailing the mechanism of action and efficacy summary. Additionally, these agents may be inaccessible, as candidacy and cost remain barriers to implementation. As our understanding of the pathophysiology and immunologic players in the persistence of AD continue to evolve, a more personalized and inclusive approach to treatment can be adopted.

## Figures and Tables

**Table 1:** Emerging therapeutics in AD and mechanisms of action. (+/++/+++: correspond to various levels of efficacy, with + indicating minimal efficacy.)

Therapeutic	Mechanism of Action	Efficacy Summary	Key Studies
Dupilumab	Inhibits IL-4 and IL-13 signaling through competitive binding to the shared subunit of the IL-4 receptor, leading to gene expression changes that normalize skin condition.	+++ (High efficacy demonstrated in numerous randomized control trials with significant improvements in IGA and EASI scores, along with patient-reported outcomes. Comparable effectiveness between dosing frequencies.)	SOLO 1 and SOLO 2 [38,39] LIBERTY AD CHRONOS Trial [40] LIBERTY AD ADOL Trial [41]
Tralokinumab	Targets and binds strongly to IL-13, inhibiting the inflammatory processes associated with AD.	++ (Improvement in IGA scores and EASI-75 in phase 3 trials. Maintenance of efficacy up to week 52 demonstrated, with AEs including infections and headaches.)	ECZTRA 1 [46] ECZTRA 2 [46] ECZTRA 6 [47]
Lebrikizumab	Directly targets IL-13, interrupting the signaling pathway responsible for inflammation and potentially restoring the skin’s natural barrier function.	+++ (Significant improvement in IGA scores and EASI-75 responses in trials. High efficacy in reducing pruritus and improving sleep among patients, with noted AEs such as conjunctivitis and dermatitis exacerbation.)	Advocate1 Trial [50] Advocate 2 Trial [50]
Nemolizumab	Targets and inhibits IL-31RA, disrupting the IL-31 signaling pathway associated with the itch-scratch cycle in AD.	++ (Significant reductions in pruritus scores and improvements in skin lesions and disease severity reported. Mild-to-moderate AEs noted, including injection site reactions and upper respiratory tract infections.)	XCIMA Trial [53]
Rocatinlimab	Inhibits and reduces OX40 pathogenic T cells responsible for AD inflammatory responses by blocking OX40.	++ (Significant reductions in EASI score observed in phase IIb study, with progressive and maintained improvements post-treatment. Common AEs include pyrexia, nasopharyngitis, and chills.)	[54]
Janus Kinase Inhibitors (JAKi)	Inhibits JAK-STAT signaling pathway, crucial for the cytokine-mediated communication involved in AD.	+++ (Significant improvement in AD symptoms and disease measures in clinical trials, highlighting efficacy in both pediatric and adult populations with manageable safety profiles.)	BREEZE-AD PEDS Clinical Trial [61]
		Baricitinib	BREEZE-AD4 Trial [62]
		Upadacitinib	The AD Up Trial [63]
		Ruxolitinib	The TRuE-AD Trial [64]
		Abroctinib	The JADE MONO 1 and JADE MONO 2 [65,66]
Crisaborole	Selective inhibitor of PDE4, reducing inflammation by inhibiting the breakdown of cyclic adenosine monophosphate.	++ (Effective in reducing AD symptoms with a good safety profile, primarily involving application site reactions. Demonstrated efficacy in both adult and pediatric patients.)	CrisADe CARE 1 [27] AD-301 Trial [28] AD-302 Trial [28] [23,26,29]
Roflumilast	Second-generation PDE4 inhibitor, reducing inflammation with a reduced risk of causing emesis compared to predecessors.	+ (Showed minimal effectiveness in AD treatment in clinical trials, with AEs including application site pain and gastrointestinal symptoms.)	[22]
Difamilast	PDE4 inhibitor that reduces cytokine production associated with inflammatory conditions like AD.	+++ (Significant improvements in AD severity and symptoms with a well-tolerated safety profile in both adult and pediatric patients.)	[35,36]
Asivatrep (TRPV1 Antagonist)	Inhibits TRPV1, reducing pruritus and inflammation associated with AD.	++ (Demonstrated effectiveness in improving clinical signs and symptoms of AD with a well-tolerated safety profile.)	CAPTAIN-AD Trial [67]
Tapinarof (Aryl Hydrocarbon Receptor Antagonist)	Modulates the aryl hydrocarbon receptor signaling pathways, reducing proinflammatory cytokine expression and oxidative stress.	++ (Showed promising efficacy and safety in treating AD, with improvements in skin clarity and reduced inflammation in clinical trials.)	[71,72]

## Data Availability

Not applicable.
